# Enhancing Global Health Security in Sub-Saharan Africa: The case for integrated One Health surveillance against zoonotic diseases and environmental threats^☆^

**DOI:** 10.1016/j.onehlt.2025.101136

**Published:** 2025-07-09

**Authors:** Pierre Gashema, Placide Sesonga, Patrick Gad Iradukunda, Richard Muvunyi, Jean Claude Mugisha, Jerome Ndayisenga, Tumusime Musafiri, Richard Habimana, Radjabu Bigirimana, Alice Kabanda, Misbah Gashegu, Noel Gahamanyi, Jonathan Izudi, Emmanuel Edwar Siddig, Jean Claude Semuto Ngabonziza, Ayman Ahmed, Tafadzwa Dzinamarira, Leon Mutesa, Claude Mambo Muvunyi

**Affiliations:** a Rwanda Joint Task Force for Marburg Virus Disease Outbreak, Ministry of Health, Rwanda Biomedical Centre, Kigali, Rwanda; b Africa Centres for Disease Control and Prevention, Addis Ababa, Ethiopia; c Research Department, Repolicy Research Centre, Kigali, Rwanda; d Division of Clinical Medicine, University of global health Equity, Kigali, Rwanda; e Drugs Department, Rwanda Food and Drugs Authority, Kigali, Rwanda; f Conservation Department, Rwanda Development Board, Kigali, Rwanda; g Partners in Health, Kigali, Rwanda; h Food and Agriculture Organisation of the United Nations,Rwanda; i Rwanda Biomedical Centre, Kigali, Rwanda; j Department of Community Health, Faculty of Medicine, Mbarara University of Science and Technology, Mbarara, Uganda; k Department of Clinical Biology, University of Rwanda, Kigali, Rwanda; l School of health Systems and Public Health, University of Pretoria, South Africa; m Center for Human Genetics, College of Medicine and Health Sciences, University of Rwanda, Kigali, Rwanda

**Keywords:** One health, Africa, Zoonotic, Surveillance, Health security, Ecosystems

## Abstract

Integrated One Health surveillance is pivotal to Africa's future health security, particularly in preventing and managing zoonotic and environmental health threats. The One Health strategy recognizes the interconnectedness of human, animal, and environmental health, allowing a holistic framework for tracking and responding to emerging and re-emerging pathogens. The One Health approach facilitates cross-sectoral data sharing and enhances surveillance, enabling the early detection and response to potential outbreaks. This proactive approach shifts the paradigm from reactive crisis management to preventive containment strategies. However, challenges such as funding gaps, limited infrastructure, limited diagnostic capacity, and weak multi-sectoral and cross-border collaborations remain. This perspective paper aims to 1) explore the effectiveness of integrated One Health surveillance in early detection and response to zoonotic diseases and environmental threats in Sub-Saharan Africa (SSA), and 2) identify key challenges and proposed solutions to strengthen regional health security. A multisectoral laboratory working group (MLWG) emerged as a pillar to enable active surveillance targeting humans, animals, and the environment. This paper highlighted essential strategies for enhancing One Health surveillance in SSA in light of the recent Marburg virus disease in Rwanda. It emphasizes environmental sampling through animal excreta and wastewater surveillance for early zoonotic detection, advocates for point-of-care polymerase chain reaction (PCR) testing platforms, and multiplex models to improve decentralized diagnostics. With 48 % of African nations incorporating One Health in national agendas, a unified continental framework is needed to support broader adoption and advance regional health security.

## 1 Introduction

Africa's high risk for zoonotic disease outbreaks is shaped by close human-animal interactions, rich biodiversity, and ecological disruptions, creating favorable conditions for pathogens to spill over from animals to humans [1]. Diseases like Ebola and Marburg frequently emerge from wildlife reservoirs, with initial transmission confirmed as animal-to-human [[2], [3], [4]]. Approximately 75 % of emerging infectious diseases are zoonotic, often linked to ecosystem disruptions, highlighting the urgent need for surveillance [5]. Africa's vulnerability to these threats is further heightened by climate change, emphasizing the need for integrated approaches to monitor and mitigate zoonotic risks effectively [5,6]. The Ebola outbreak in West Africa from 2014 to 2016 was indeed traced back to an animal origin, specifically fruit bats, which are believed to be the natural reservoir for the virus [7]. The outbreak began in Guinea and spread to several neighboring countries, resulting in significant public health challenges [7]. The Marburg outbreak in Uganda was linked to animals [8]. Similarly, the ongoing Marburg virus disease outbreak in Rwanda originated from contact with animals [9]. Other past zoonotic disease outbreaks within the East African region, namely Rift Valley fever and Mpox have also been traced to have origins in animals hence underscoring the importance of integrating animal, human, and environmental health (One Health) in outbreak surveillance, detection, and response [10,11]. This risk is enhanced by rapid population growth and cross-border movements, unplanned urbanization, industrialization, and changes in land use and cover thus driving people into previously undisturbed habitats, intensifying contact between humans, domestic animals, and wildlife, including the emergence and spread of invasive diseases [12,13]. The effectiveness of one health approach was emphasized during the Mpox and Marburg response as a key strategy for outbreak containment [14,15]. A One Health prioritization exercise was implemented recently in Rwanda and it has identified several zoonotic diseases of public health importance in the country [16]. This includes zoonotic bacterial infections like zoonotic tuberculosis, Brucellosis, and Salmonellosis and parasitic diseases such as Toxoplasmas and Trypanosomiasis. Interestingly, the top priority zoonotic viral infections included Rabies, Influenza, and viral hemorrhagic fever such as Ebola, Marburg virus, Rift Valley fever, and Yellow fever [17]. Compounding Africa's vulnerability to zoonotic diseases is the weak health system, limited capacity of healthcare infrastructure, low staffing or human resources for health, inadequate healthcare financing, weak medical logistics and supply chain system, weak diagnostic capacity, and inadequate data and information used in many countries [18]. This limitation hampers the ability to rapidly detect outbreaks and respond swiftly [19]. Resource constraints, combined with inadequate mechanisms for real-time data sharing across sectors such as animal, human, environment, education, and security among others often lead to fragmented and ineffective responses, exacerbating the public health crisis when outbreaks occur [[5], [20]].

Environmental stressors including pollution, deforestation, global warming, and water scarcity further weaken the immune resilience of populations, making them more susceptible to zoonotic infections [21].

Fragmented surveillance, with human, animal, and environmental health sectors working in silos, hinders countries' ability to develop cost-effective policies and action plans for disease outbreaks. [22]. In this fragmented model, vital data rarely circulates promptly, delaying evidence gathering to guide policymaking, strategic planning, and disease detection and control [23]. The limitations of traditional surveillance systems include data standardization issues, low capacity leading to underestimation of disease severity, and predefined target pathogens [24]. The lack of an integrated, multi-sectoral, One Health system for surveillance, preparedness, prevention, and response to epidemics and pandemics significantly hampers timely detection of zoonotic threats, coordinated action across human, animal, and environmental health sectors, and the effective containment of emerging infectious diseases. Furthermore, it restricts the capacity of countries and undermines policy and decision-making, including strategic planning. This reduces the cost-effectiveness of interventions, leading to greater health and socioeconomic impacts, particularly among vulnerable communities. All these factors and challenges underscore the need for strengthening Global Health Security at sub-national, national, and regional levels through a One Health lens [25,26]. This policy perspective aims to strengthen One Health surveillance in Africa by endorsing environmental sampling and decentralized diagnostics, leveraging emerging local manufacturing capacities in Africa. Additionally, it calls for a unified continental framework to support the integration of One Health in national policies, enhancing health security across the region.

## 2 Materials and methods

A targeted literature search was conducted to identify relevant peer-reviewed articles and official reports related to zoonotic disease surveillance, One Health approaches, and outbreak response in Africa. The search covered publications from January 1, 2015 to December 31, 2024, using databases including PubMed, Scopus, and Google Scholar. Search terms included combinations of “One Health,” “zoonotic diseases,” “Africa,” “surveillance,” “outbreak response,” “Marburg,” “Ebola,” and “Mpox”.

Inclusion criteria consisted of articles in English that focused on zoonotic disease emergence, One Health surveillance implementation, and policy-related frameworks in African contexts. Exclusion criteria included articles without clear relevance to human-animal-environmental health intersections, editorials without supporting data, or studies conducted outside the African region. Policy recommendations extracted from government and institutional reports such Africa CDC and WHO were also included where appropriate. Duplicates and non-peer-reviewed materials were excluded. In total, approximately 80 documents were screened, and 55 sources were selected for inclusion. The information was analyzed thematically and synthesized to inform the key policy perspectives discussed in this article. All included sources are cited in the reference list.

## 3 Rationale for One Health surveillance in Africa

**Fig. 1:**
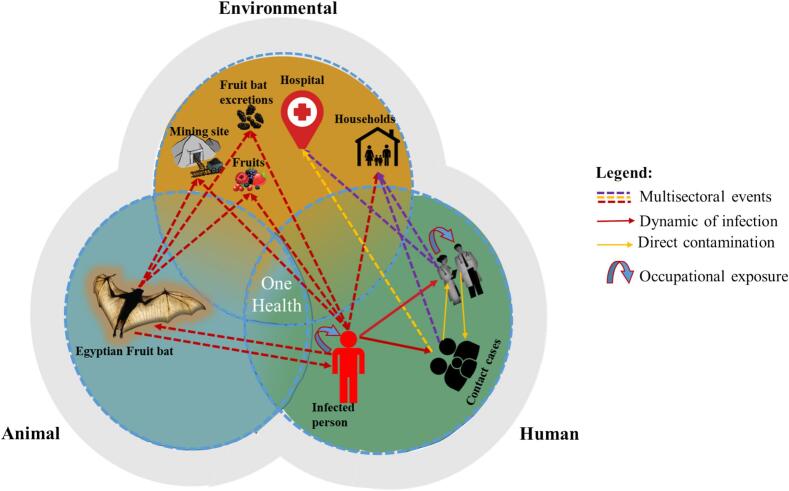
The figure illustrates the One Health surveillance to the recent MDV in Rwanda, demonstrating how the infection spread from fruit bats at a mining site to humans in a single jump [3,9,28]. The index case spread the virus to healthcare providers and probably household members. Furthermore, we demonstrate how the outbreak was monitored among the contacts. MDV thus demonstrates the interconnection between animals, humans, and the environment and underlines the importance of cross-sector collaboration in addressing zoonotic diseases.

Growing evidence indicates interconnectivity and co-dependency between human, animal, and environmental health. This improved understanding is currently adopted and translated by the National Public Health Institute such as the Rwanda Biomedical Center and their regional stakeholders like the African Centers for Diseases Prevention and Control. Jointly, they safeguard the Global Health Security in the region through an integrated multi-sectoral One Health strategy that goes beyond working in silos [10,27]. Recent outbreaks, such as Ebola, Marburg, and Mpox, serve as critical reminders of this interdependence. Each of these viruses has its origins in animal populations, primarily zoonotic reservoirs like fruit bats (Fig. 1) and primates before spilling over to humans.

This zoonotic transmission is increasingly fueled by anthropogenic activities like deforestation, urbanization, and heightened human-animal interactions resulting from changes in land use and wildlife trade [29]. The primary zoonotic spillover of Marburg Virus Disease (MDV) in Rwanda occurred in a naïve population as no prior exposure to the virus was reported [9]. The regional presence of a zoonotic virus which can lead to a severe outbreak or pandemic should trigger surveillance at the regional level.

The necessity for integrated disease surveillance across human, animal, and environmental sectors is paramount in facilitating the rapid detection of infectious disease outbreaks, a strong surveillance system significantly contributes to disease control through improved early detection and response. Adopting One Health surveillance, we improve the disease prevention, detection and response to public health, economies, and the entire ecosystem. Moreover, One Health surveillance fosters collaboration among veterinarians, medical professionals, ecologists, and policymakers among others hence creating a comprehensive understanding of health threats. This collaboration is instrumental in developing targeted interventions, informing public health policies, and promoting sustainable practices that protect both human and animal health.

## 4 Progress of adapting One Health surveillance to address zoonotic pathogens and antimicrobial resistance in Africa

**Fig. 2:**
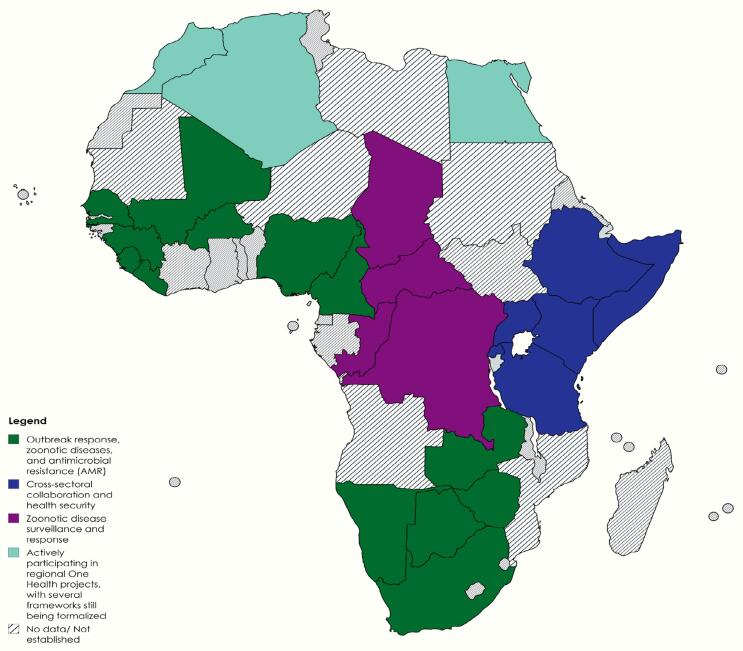
Map showing progress made within different regions: Countries already integrated and countries formalizing the approach.

Only 48 % of African nations have incorporated the One Health strategy into their action plans, indicating slower regional growth, compared to 54 % of nations worldwide that have done so [30]. Nonetheless, 26 African countries have adopted One Health principles, demonstrating the continent's dedication to tackling interrelated health hazards in the human, animal, plant, crops, and environmental domains [30]. As shown in Fig. 2, the Western Africa regions have set up One Health platforms or strategic plans that address outbreak response, zoonotic illnesses, and antimicrobial resistance [31]. Within Eastern Africa, the use of One Health surveillance has led to comparable strategic action plans to improve cross-sectoral cooperation and health security [32]. In northern Africa, several One Health surveillance countries are still formalizing their frameworks and are actively involved in regional One Health projects [25]. For example, the One Health initiative is being carried out in Central Africa with a focus on zoonotic disease surveillance and response [33]. Southern Africa regions have focused on having national plans for pandemic preparedness, antimicrobial resistance, and health security [34]. These varied initiatives show a robust, well-coordinated regional response to health issues affecting several countries.

## 5 Early detection of emerging and re-emerging zoonotic pathogens and rapid action

**Fig. 3:**
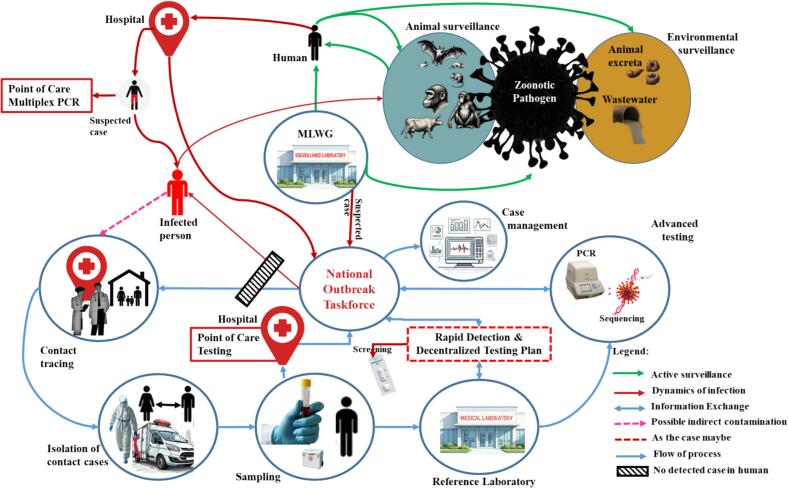
Demonstrates the active zoonotic virus surveillance in a community which should be established continuously through Animal (bats, rodents, primates, and cows), Environmental (sampling animal excreta and wastewater), and human Surveillance concerning zoonotic pathogens in the regional context. The MLWG serves as a cornerstone for detecting potential hazards a priori.

One Health surveillance fosters early detection by enabling systematic and timely data collection across public, veterinary, and environmental health sectors [35]. A multisectoral laboratory working group (MLWG) was recommended as a pillar to enable active surveillance of humans, animals, and the environment [36]. Prioritizing individualized multiplex PCR testing with a platform that can screen a variety of diseases and bolster regional surveillance, particularly in animals to minimize spillover incidents, is one way to fortify the working group [37]. Fig. 3 highlights the need for continuous surveillance of viruses in Africa, emphasizing the significance of investing in research infrastructures.

An integrated One Health data system will enhance evidence generation, decision-making, and understanding of concerns hence facilitating immediate outbreak response, saving lives and resources, and enhancing the overall health outcomes [38]. In Uganda, for instance, a multisectoral One Health surveillance successfully detected an Ebola spillover from animals to humans, enabling prompt interventions that contained the outbreak [39]. Adequate response to Ebola in Uganda was empowered by lessons from COVID-19 containment whereby up to six weeks of lockdown were employed. More importantly, the repurposing of infrastructure used in the COVID-19 response such as incident command stimulated adequate response mechanisms at all levels [40]. Hypothetical models demonstrate that coordinated surveillance of Avian Flu in poultry prevents escalation to human populations, underscoring the critical role of integrated surveillance in outbreak detection, response, and prevention [41]. Effective monitoring of emerging and re-emerging infectious diseases at both health facility and community levels demands robust coordination and rapid intervention within routine surveillance systems. Therefore, integrating Point-of-care (POC) multiplex PCR testing is excellent, as it enables immediate local action. Even though we are advocating for centralized surveillance management, it is important to acknowledge that data from lower-level surveillance sites are essential. POC testing supports this by facilitating timely data collection, accurate recording and reliable transmission systems to ensure effectiveness [42]. In the African continent, the successful implementation of point-of-care diagnostic tools requires continuous education and training programs, reliable supply chains, and the involvement of private and public partnerships [43].

## 6 Use of Artificial Intelligence (AI) in One Health surveillance

AI plays a pivotal role in One Health surveillance, offering innovative solutions for early antimicrobial resistance detection, optimized antibiotic use, and accelerated drug discovery [44]. In Europe, platforms like the Epi Connect Intelligence Platform enhance real-time data sharing, adhering to findable, accessible, interoperable, and reusable data to improve epidemiological analysis and outbreak response [45]. Integrating AI with emerging technologies and national public health systems enhances collaborative, data-driven surveillance, enabling proactive risk assessment and the development of policies to combat antimicrobial resistance and protect global health.

## 7 Challenges to implementing One Health surveillance in Africa

Implementing One Health surveillance in Africa faces challenges that limit effective disease detection and response across human, animal, and environmental sectors. For example, limited resource availability resulting from inadequate funding to the healthcare system has led to a weak laboratory and diagnostic capacity and a shortage of trained personnel, hindering the establishment of robust surveillance systems for zoonotic pathogens [34,46,47]. The lack of practical experience among stakeholders involved in One Health surveillance implementation is another major barrier in Africa, as it may slow epidemic response [48]. Traditional medicines, insufficient legal frameworks, and lack of government support hinder One Health Surveillance implementation [49]. Siloed structures have created inefficiency, even with progress in multisectoral One Health coordination in some countries [49]. The successful implementation of One Health in Africa faces a range of challenges. These include environmental factors such as soil health, land degradation, biodiversity loss, and climate change; health-related concerns such as nutrition and food security, water, sanitation and hygiene, food safety, and antimicrobial resistance; and social issues like gender equity [50]. Overall, the effective implementation of One Health surveillance needs prioritization [51].

## 8 Recommendations for policy and practice

### 8.1 Strengthening the establishment of MLWG in SSA

Africa Centers for Disease Control and Prevention established a multi-sectoral One Health surveillance at the country level emphasizing the need to establish the MLWG but the implementation has not started as MLWG has not been established in most countries in SSA. Moreover, MLWG serves as a center of active One-Health surveillance (Fig. 3), therefore, there is a need to facilitate its operationalization. The WHO/Europe established the Multisectoral National Laboratory Working Groups (NLWGs) to develop national health laboratory priorities and policies. These groups support the implementation of One Health surveillance and coordinate international donors to strengthen laboratory medicine capacity [52]. This paper offers a practical recommendation for integrating NLWGs into SSA to enhance One Health surveillance.

### 8.2 Establishing a regular National Outbreak Task Force Committee

SSA countries should establish a committee to assess MLWG reports, with a team ready to deploy in case of a suspicious pathogen in communities, environments, or animals. This task force should meet regularly and has been used during outbreaks like COVID-19 or potential border threats, such as the National Ebola Task Force in Rwanda despite zero cases. This will enable early surveillance and the effective deployment of outbreak response teams, who are trained and experienced from previous pandemic and outbreak responses. The Africa Task Force for Novel Coronavirus was established to coordinate preparedness and response efforts for the global 2019 Novel Coronavirus epidemic [53] and serve as a model for national levels for mainly reporting to MLWG.

### 8.3 Promoting research infrastructures to drive the manufacturing of cost-effective personalized point-of-care multiplex PCR platforms

Effective zoonotic disease surveillance relies on infrastructure. Initiatives like the Africa Medicines Agency, which aims to enhance research capacity, should be prioritized to develop context-specific solutions. Point-of-care Multiplex PCR and MLWG would ensure early detection of zoonotic pathogens.

### 8.4 Effective coordination of multisectoral One-health coordination mechanisms

In Africa, more countries are adopting multisectoral One Health coordination, though outbreak responses still tend to work in silos. Countries like Rwanda and Uganda have made notable progress with the One Health approach, but empowering such mechanisms across all of SSA would break silos and improve early outbreak detection.

### 8.5 Funding and capacity building for effective implementation and sustainability of multisectoral One-health coordination mechanisms

Capacity building for multisectoral One Health coordination should target all stakeholders. A contextualized One Health training curriculum would support multisectoral One Health coordination implementation at national and regional levels. The Africa Pathogen Genomic Initiative framework serves as an example of such a training package, though it is primarily used during outbreaks and in high-burden vector disease contexts. Sufficient funding and effective fund management are essential, with funding mechanisms embedded in national One Health action plans. African Union member states must be involved to ensure full implementation of One Health surveillance systems.

## 9 Future consideration for health authorities and governments in Africa

To advance One Health surveillance in Africa, governments must prioritize a unified continental framework for cross-border collaboration and data sharing. Strengthening laboratory networks, including MLWGs, will enhance diagnostics and enable real-time pathogen tracking across health sectors. Sustainable funding should support decentralized diagnostics, such as point-of-care PCR and multiplex testing, in remote, high-risk areas. Additionally, integrating environmental surveillance like wastewater and animal excreta testing can serve as an early warning system for zoonotic threats. Aligning policies between African nations and fostering public-private partnerships will ensure resilient health systems and proactive containment of emerging diseases.

## 10 Conclusion

This perspective enhances our understanding of One Health surveillance in SSA, emphasizing systematic surveillance at the human, animal, and environmental health interface. Deployment of multiplex PCR platforms at surveillance sites and point-of-care testing is urgently needed for early outbreak diagnosis, response, and control. Health interventions based on multisectoral One-health coordination mechanisms and MLWGs should be prioritized to ensure effective response and fund management in SSA. This would enable African states to implement One Health surveillance for outbreak investigation and break siloed working, ensuring regional health security.

## Authors statements

All authors, affirm that this manuscript is an original work and has not been published or submitted for publication elsewhere. All authors have significantly contributed to the conception, design, analysis, and drafting of the manuscript and approve its final version for submission.

## CRediT authorship contribution statement

**Pierre Gashema:** Writing – review & editing, Writing – original draft, Investigation, Conceptualization. **Placide Sesonga:** Writing – original draft, Investigation, Formal analysis. **Patrick Gad Iradukunda:** Visualization, Validation, Formal analysis, Data curation, Conceptualization. **Richard Muvunyi:** Writing – review & editing, Investigation, Formal analysis. **Jean Claude Mugisha:** Writing – review & editing, Visualization, Investigation, Conceptualization. **Jerome Ndayisenga:** Writing – review & editing, Validation, Methodology, Formal analysis. **Tumusime Musafiri:** Writing – review & editing, Visualization, Formal analysis, Conceptualization. **Richard Habimana:** Writing – review & editing, Visualization, Validation, Resources, Investigation. **Radjabu Bigirimana:** Writing – review & editing, Supervision, Project administration, Conceptualization. **Alice Kabanda:** Writing – review & editing, Methodology, Investigation, Conceptualization. **Misbah Gashegu:** Writing – review & editing, Visualization, Conceptualization. **Noel Gahamanyi:** Writing – review & editing, Visualization, Validation, Data curation. **Jonathan Izudi:** Writing – review & editing, Visualization, Validation, Conceptualization. **Emmanuel Edwar Siddig:** Writing – review & editing, Writing – original draft, Validation, Conceptualization. **Jean Claude Semuto Ngabonziza:** Writing – review & editing, Visualization, Formal analysis, Conceptualization. **Ayman Ahmed:** Writing – review & editing, Writing – original draft, Methodology, Conceptualization. **Tafadzwa Dzinamarira:** Writing – review & editing, Visualization, Validation, Supervision, Formal analysis, Conceptualization. **Leon Mutesa:** Writing – review & editing, Visualization, Validation, Project administration, Conceptualization. **Claude Mambo Muvunyi:** Writing – review & editing, Writing – original draft, Validation, Supervision, Project administration, Conceptualization.

## Funding statement

This research received no specific grant from any funding agency in the public, commercial, or not-for-profit sectors.

## Declaration of competing interest

All authors declared that there is no any conflict of interest including employment, consultancies, funding and others competing interest.

## Data Availability

No data was used for the research described in the article.
